# Bromocriptine treatment and outcomes in peripartum cardiomyopathy: the EORP PPCM registry

**DOI:** 10.1093/eurheartj/ehae559

**Published:** 2024-09-02

**Authors:** Peter van der Meer, Bart Johan van Essen, Charle Viljoen, Michael Böhm, Alice Jackson, Denise Hilfiker-Kleiner, Julian Hoevelmann, Alexandre Mebazaa, Hasan Ali Farhan, Sorel Goland, Wouter Ouwerkerk, Mark C Petrie, Petar M Seferović, Jasper Tromp, Karen Sliwa, Johann Bauersachs

**Affiliations:** Department of Cardiology, University of Groningen, University Medical Centre Groningen, Groningen, The Netherlands; Department of Cardiology, University of Groningen, University Medical Centre Groningen, Groningen, The Netherlands; Cape Heart Institute, Faculty of Health Sciences, University of Cape Town, Cape Town, South Africa; Division of Cardiology, Department of Medicine, Groote Schuur Hospital, Faculty of Health Sciences, University of Cape Town, Cape Town, South Africa; Department of Internal Medicine III-Cardiology, Angiology, and Internist Intensive Medicine, Saarland University Hospital, Homburg, Saar, Germany; British Heart Foundation Cardiovascular Research Centre, University of Glasgow, Glasgow, UK; Philipps-Universität Marburg, Medical Faculty, Marburg, Germany; Cape Heart Institute, Faculty of Health Sciences, University of Cape Town, Cape Town, South Africa; Department of Internal Medicine III-Cardiology, Angiology, and Internist Intensive Medicine, Saarland University Hospital, Homburg, Saar, Germany; Paris Cité University, French National Institute of Health and Medical Research (INSERM) Cardiovascular MArkers in Stress Conditions (MASCOT), Paris, France; Department of Anesthesiology and Critical Care, Saint Louis Lariboisière Hospitals, Public Assistance Hospital of Paris, Paris, France; Iraqi Board for Medical Specialisations, University of Baghdad, College of Medicine, Baghdad, Iraq; Kaplan Medical Center, The Heart Institute, Rehovot, Israel; Israel Hadassah Medical School, Hebrew University, Jerusalem, Israel; Amsterdam UMC, University of Amsterdam, Amsterdam Infection and Immunity Institute, Amsterdam, The Netherlands; British Heart Foundation Cardiovascular Research Centre, University of Glasgow, Glasgow, UK; Faculty of Medicine, University Medical Center, Belgrade, Serbia; Serbian Academy of Sciences and Arts, Medical Faculty University of Belgrade, Belgrade, Serbia; Saw Swee Hock School of Public Health & The National University Health System, Singapore, Singapore; Duke-NUS Medical School, Singapore, Singapore; National Heart Centre Singapore, Singapore, Singapore; Cape Heart Institute, Faculty of Health Sciences, Department of Medicine and Cardiology, University of Cape Town, Cape Town, South Africa; Department of Cardiology and Angiology, Hannover Medical School, Hannover, Germany

**Keywords:** Peripartum cardiomyopathy, Bromocriptine, Pregnancy, Heart failure

## Abstract

**Background and Aims:**

Peripartum cardiomyopathy (PPCM) remains a serious threat to maternal health around the world. While bromocriptine, in addition to standard treatment for heart failure, presents a promising pathophysiology-based disease-specific treatment option in PPCM, the evidence regarding its efficacy remains limited. This study aimed to determine whether bromocriptine treatment is associated with improved maternal outcomes in PPCM.

**Methods:**

Peripartum cardiomyopathy patients from the EORP PPCM registry with available follow-up were included. The main exposure of this exploratory non-randomized analysis was bromocriptine treatment, and the main outcome was a composite endpoint of maternal outcome [death or hospital readmission within the first 6 months after diagnosis, or persistent severe left ventricular dysfunction (left ventricular ejection fraction < 35%) at 6-month follow-up]. Inverse probability weighting was used to minimize the effects of confounding by indication. Multiple imputation was used to account for the missing data.

**Results:**

Among the 552 patients with PPCM, 85 were treated with bromocriptine (15%). The primary endpoint was available in 491 patients (89%) and occurred in 18 out of 82 patients treated with bromocriptine in addition to standard of care (22%) and in 136 out of 409 patients treated with standard of care (33%) (*P* = .044). In complete case analysis, bromocriptine treatment was associated with reduced adverse maternal outcome [odds ratio (OR) 0.29, 95% confidence interval (CI) 0.10–0.83, *P* = .021]. This association remained after applying multiple imputation and methods to correct for confounding by indication (inverse probability weighted model on imputed data: OR 0.47, 95% CI 0.31-0.70, *P* < 0.001). Thromboembolic events were observed in 6.0% of the patients in the bromocriptine group vs. 5.6% in the standard of care group (*P* = .900).

**Conclusions:**

Among women with PPCM, bromocriptine treatment in addition to standard of care was associated with better maternal outcomes after 6 months.

## Structured Graphical Abstract

**Structured Graphical Abstract:**
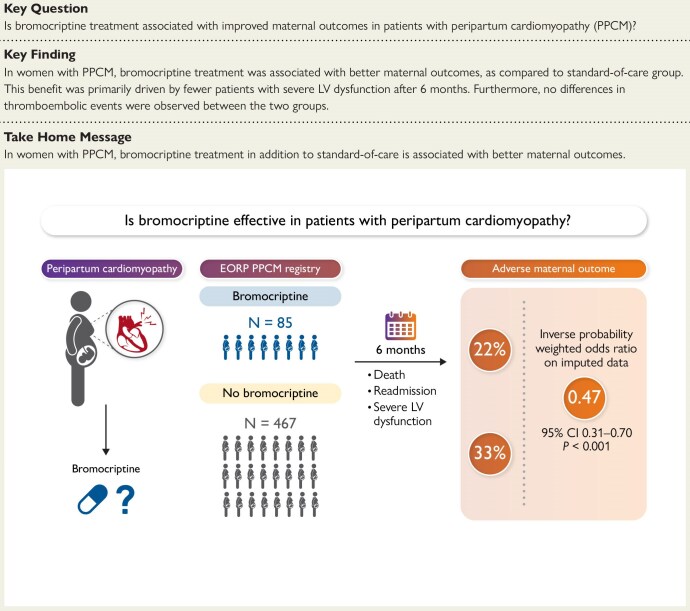
The association between bromocriptine treatment and maternal outcome in patients with peripartum cardiomyopathy. CI, confidence interval; LV, left ventricular.

**See the editorial comment for this article ‘Bromocriptine in the treatment of peripartum cardiomyopathy: is it ready for prime time?', by U. Elkayam, https://doi.org/10.1093/eurheartj/ehae875.**

## Introduction

Peripartum cardiomyopathy (PPCM) is a significant cause of global maternal mortality, particularly in some regions.^1^ It is defined as a cardiomyopathy with left ventricular ejection fraction (LVEF) < 45% that occurs late during pregnancy or in the early postpartum period.^2,3^ While the disease course may be mild in some cases, mortality rates reach as high as 20%, despite the young age of the patients.^4^ Recently, a simple score was derived from the EORP PPCM registry to predict left ventricular (LV) recovery in patients with PPCM.^5^ Although guideline-directed medical therapy (GDMT) for heart failure with reduced ejection fraction is recommended in patients with PPCM, the disease lacks proven disease-specific therapies.^1^

From a mechanistic perspective, oxidative stress in combination with elevated concentrations of prolactin results in the generation of a pro-apoptotic 16 kDa prolactin fragment.^6^ Dopamine 2D receptor agonists, such as bromocriptine, are prolactin-suppressing agents used to cease nursing and have successfully attenuated PPCM in mouse models^6,7^ and have therefore been investigated as a potential treatment of PPCM, yet existing evidence remains limited.^1,8,9^ Regional disparities persist in recommendations regarding its use, with no consensus reached in the USA while the European Society of Cardiology (ESC) guideline suggests that it may be considered (IIb recommendation).^10,11^ The ongoing Randomized Evaluation of Bromocriptine in Myocardial Recovery Therapy for Peripartum Cardiomyopathy (REBIRTH) trial aims to address this uncertainty, but results are not expected until 2029, underscoring the need for additional evidence to inform treatment decisions in PPCM (ClinicalTrials.gov number, NCT05180773).

In this prospective, multicentre, cohort study, we aim to bridge this gap in evidence by investigating the association between bromocriptine treatment and adverse maternal outcomes 6 months post-diagnosis in women with PPCM.

## Methods

In 2011, the ESC invited over 100 national and affiliated member cardiac societies to contribute to a global registry on PPCM as part of the ESC EURObservational Research Programme (EORP).^12^ These societies were tasked with identifying centres to participate in the registry. In low-income countries without national cardiac societies, potential study sites were identified through abstracts and publications. Participating centres were required to have clinical expertise in diagnosing PPCM, availability of echocardiography, and the ability to follow up with patients for at least 6 months.

Women newly diagnosed with PPCM were enrolled in the study between 2012 and 2018 with a mandatory 6-month follow-up. To be eligible, participants had to meet the following criteria at the time of diagnosis: (i) be in a peripartum state (i.e. last trimester of pregnancy up to the first 6 months postpartum), (ii) exhibit signs and/or symptoms of heart failure, (iii) have an LVEF ≤45%, and (iv) have no other identifiable causes of heart failure. From the initial registry of 739 patients,^13^ only those with known information regarding their bromocriptine therapy status (*n* = 552) were included in this study.

Baseline data collected included demographics, comorbidities, obstetric history, signs, symptoms, and results from blood tests, electrocardiography, chest radiography, echocardiography, and pharmacological therapy.

Poor outcome was determined by the composite endpoint of death; hospital readmission, within the first 6 months after diagnosis; or persistent severe LV dysfunction (LVEF < 35%) at 6-month follow-up. Secondary outcomes included thromboembolic events (either arterial or venous).

The study complied with the Declaration of Helsinki, with investigators participating voluntarily and without compensation. Approvals were obtained by participating centres from national or regional ethics committees and Institutional Review Boards, following local regulations. This observational study was supported by a central study management team and did not involve specific protocols or recommendations for diagnosis or management.

### Statistical analysis

Descriptive statistics were used to summarize the data. Continuous variables were presented as means with standard deviations (SD) for parametric data or medians with interquartile ranges (IQR) for non-parametric data. Categorical variables were expressed as frequencies and percentages. Baseline characteristics were described and stratified based on whether patients received bromocriptine treatment. Continuous data were compared using independent *t*-tests for normally distributed data or the Mann–Whitney *U* test for non-normally distributed data. Binary data were compared using Pearson’s *χ*^2^ test or Fisher’s exact test depending on the sample size.

### Multiple imputation and inverse probability weighting

Missing data was imputed with the Multivariate Imputation with Chained Equations (mice, version 3.14.0) in R. Fifty imputed data sets were generated with five iterations per data set. Continuous variables were imputed with predictive mean matching, binary data with logistic regression, and categorical data with polytomous regression. No abnormalities in the imputed values were found.

Due to the non-randomized nature of this study, there is the risk of confounding by indication. To minimize the potential impact of this bias, inverse probability weighting and multivariable adjusted regression were used. In short, first, least absolute shrinkage and selection operator (LASSO) regression was used to identify variables related to adverse maternal outcome. The R package glmnet (version 4.1–4) was used to perform LASSO regression, and directed acyclic graphs were constructed to identify possible confounders for the association between bromocriptine treatment and adverse maternal outcome. Due to multicollinearity between LV end-diastolic and end-systolic diameter, only LV end-diastolic diameter (LVEDD) was included. History of diabetes mellitus, anticoagulant treatment, treatment with mineralocorticoid receptor antagonists (MRAs), treatment with loop diuretics, systolic blood pressure at baseline, QRS duration at baseline, LVEDD, LVEF, geographical region, right ventricular failure at baseline, and cardiomegaly at chest X-ray at baseline were associated with adverse maternal outcome.

In the following step, weights were generated for inverse probability weighting according to previously described methods.^14,15^ The variables in the aforementioned paragraph were used to generate weights, except MRA, which was replaced by treatment with GDMT, which is treatment with angiotensin-converting enzyme inhibitors (ACEi) or angiotensin receptor blockers (ARBs), beta-blockers and MRAs.^13,14^ Inverse probability weighting was performed using the R package MatchThem (version 1.10). Covariate balance was assessed using the absolute standardized mean differences across all imputed data sets.

Finally, two logistic regression models were fitted, one on the weighted data and one unweighted but adjusted for multiple confounding variables.

## Results

### Baseline characteristics

**Figure 1:**
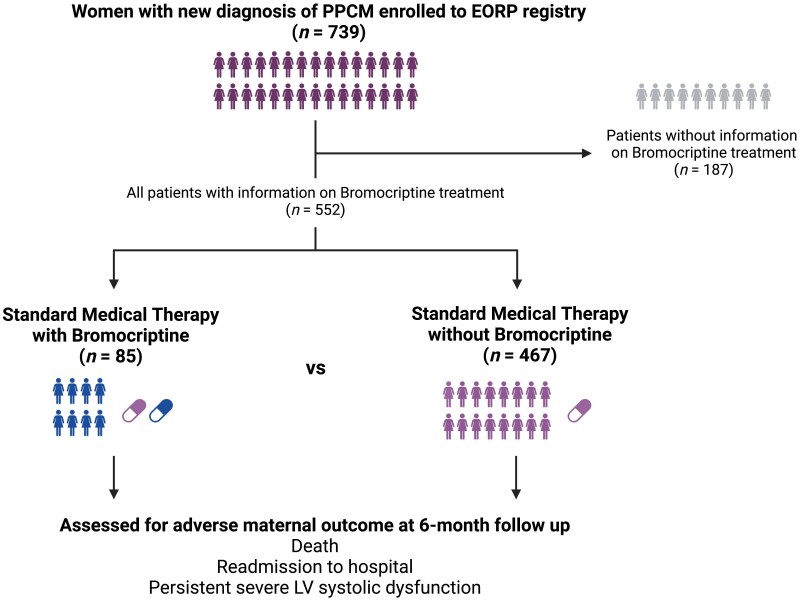
Flowchart

Of the 552 patients with PPCM, 85 received treatment with bromocriptine whereas 467 did not (*Figure 1*). There were no differences in age at baseline [bromocriptine vs. no bromocriptine: 31.5 years (± 5.6) vs. 30.9 years (± 6.2), *P* = .40], parity (>2 children 83.0% vs. 72.1%, *P* = .096), and previous presence of PPCM (7.5% vs. 7.6%, *P* = .98). There was a significant difference in the geographical region regarding bromocriptine prescription. For example, patients living in Africa were more likely to receive bromocriptine compared with other global regions (*P* < .001). Patients receiving bromocriptine experienced more severe heart failure symptoms (87.1% of women who received bromocriptine had a New York Heart Association functional class III/IV at time of diagnosis vs. 62.4% in those who did not, *P* < .001).

There were no significant differences in the presence of hypertension during pregnancy nor in LVEDD nor in QRS duration at baseline, all of which have been shown to be important predictors of LV recovery (both cohorts had a PPCM LV recovery prediction score of 4).^5^ Furthermore, no significant differences were observed in LV systolic function [LVEF 34% (IQR 26–40) in the bromocriptine group vs. LVEF 31% (IQR 23–38) in the no bromocriptine group; *P* = .09). However more patients not receiving bromocriptine had at baseline a LVEF ≤ 25% (36.2% vs. 20.2%, *P* = .014).

There were significant differences in the use of GDMT between the two groups. Patients that received bromocriptine were more likely to receive ACEi/ARBs and MRAs compared with those not receiving bromocriptine (96.5% vs. 82.9% for ACEi/ARB, *P* = .001, and 71.8% vs. 40.1% for MRA, *P* < .001). No differences in beta-blocker treatment (bromocriptine group 75.3% vs. 82.4%, *P* = .12) or oral anticoagulation at baseline were observed (bromocriptine group 20.2% vs. 15.3%, *P* = .26). Loop diuretics were significantly more often prescribed in patients receiving bromocriptine (69% vs. 98%, *P* < .001). Other baseline characteristics can be found in *Table 1*.

**Table 1:** Baseline characteristics of patients with peripartum cardiomyopathy stratified by bromocriptine treatment

	Total (*n* = 552)	No bromocriptine (*n* = 467)	Bromocriptine (*n* = 85)	*P*-value	*N*
Age (years)	31.0 ± 6.1	30.9 ± 6.2	31.5 ± 5.6	.400	549
Ethnicity				<.001	536
Asian	116 (21.6)	105 (23.2)	11 (13.1)		
Black	160 (29.9)	119 (26.3)	41 (48.8)		
Caucasian	185 (34.5)	156 (34.5)	29 (34.5)		
Hispanic	7 (1.3)	7 (1.5)	0 (0.0)		
Middle Eastern	49 (9.1)	46 (10.2)	3 (3.6)		
Other	19 (3.5)	19 (4.2)	0 (0.0)		
Region				<.001	552
Africa	165 (29.9)	122 (26.1)	43 (50.6)		
Asia-Pacific	93 (16.8)	86 (18.4)	7 (8.2)		
Europe	202 (36.6)	171 (36.6)	31 (36.5)		
Middle East	92 (16.7)	88 (18.8)	4 (4.7)		
High development index				<.001	552
High	236 (42.8)	204 (43.7)	32 (37.6)		
Medium	206 (37.3)	185 (39.6)	21 (24.7)		
Low	110 (19.9)	78 (16.7)	32 (37.6)		
Parity > 2	261 (73.7)	217 (72.1)	44 (83.0)	.096	354
Mode of delivery				.450	552
Caesarean section	262 (47.5)	226 (48.4)	36 (42.4)		
Miscarriage	5 (0.9)	5 (1.1)	0 (0.0)		
Termination	2 (0.4)	2 (0.4)	0 (0.0)		
Vaginal delivery	283 (51.3)	234 (50.1)	49 (57.6)		
Previous PPCM	27 (7.6)	23 (7.6)	4 (7.5)	.980	354
Smoking (current and former)	83 (15.6)	76 (16.9)	7 (8.8)	.066	531
Diabetes	17 (3.1)	14 (3.0)	3 (3.5)	.810	547
Hypertension during pregnancy				.530	
No hypertension	329 (60.7)	275 (59.8)	54 (65.9)		542
Hypertension without pre-eclampsia	75 (13.8)	64 (13.9)	11 (13.4)		
Pre-eclampsia	138 (25.5)	121 (26.3)	17 (20.7)		
BMI (kg/m^2^)	25.1 (22.2–29.0)	25.0 (22.2–29.2)	25.3 (22.8–27.8)	.790	534
Time between symptom onset and diagnosis (days)	11.0 (3.0–35.0)	10.5 (3.0–39.0)	11.5 (4.0–26.0)	.860	481
NYHA functional class				<.001	545
I	43 (7.9)	42 (9.1)	1 (1.2)		
II	141 (25.9)	131 (28.5)	10 (11.8)		
III	189 (34.7)	145 (31.5)	44 (51.8)		
IV	172 (31.6)	142 (30.9)	30 (35.3)		
Systolic BP (mmHg)	115.0 (100.0–134.0)	115.0 (100.0–130.0)	120.0 (100.0–140.0)	.850	535
Diastolic BP (mmHg)	79.5 (67.0–90.0)	77.0 (67.0–90.0)	80.0 (66.0–90.0)	.540	534
Heart rate (b.p.m.)	100.0 (84.0–115.0)	98.0 (82.0–112.0)	106.0 (96.0–120.0)	<.001	538
LVH	106 (20.1)	78 (17.5)	28 (33.7)	<.001	528
QTc by Bazett (ms)	453.6 (413.1–485.8)	453.0 (413.1–485.8)	458.1 (413.3–485.9)	.690	514
QRS duration (ms)	82.0 (80.0–95.0)	83.0 (80.0–96.0)	80.0 (80.0–90.0)	.340	511
Chest X-ray showing Congestion	284 (74.5)	242 (76.6)	42 (64.6)	.044	381
Chest X-ray showing cardiomegaly	303 (79.3)	245 (77.3)	58 (89.2)	.030	382
LVEDD (mm)	59.0 (54.0–64.0)	59.0 (54.0–64.0)	59.5 (55.0–64.0)	.210	513
LVESD (mm)	49.0 (45.0–54.0)	49.0 (44.0–55.0)	48.0 (45.0–54.0)	.890	446
LVEF (%)	31.0 (24.0–38.0)	31.0 (23.0–38.0)	34.0 (26.1–39.5)	.086	537
LVEF categories				.014	537
26%–35%	179 (33.3)	143 (31.6)	36 (42.9)		
>35%	177 (33.0)	146 (32.2)	31 (36.9)		
≤25%	181 (33.7)	164 (36.2)	17 (20.2)		
PPCM recovery score	4.0 (3.0–6.0)	4.0 (3.0–6.0)	4.0 (3.0–6.0)	.732	407
Beta-blocker	448 (81.3)	384 (82.4)	64 (75.3)	.120	551
ACEi/ARB	469 (85.0)	387 (82.9)	82 (96.5)	.001	552
MRA	248 (45.0)	187 (40.1)	61 (71.8)	<.001	551
Beta-blocker, ACEi/ARB and MRA	189 (34.3)	150 (32.2)	39 (45.9)	.014	551
Loop diuretic	407 (73.7)	324 (69.4)	83 (97.6)	<.001	552
Oral anticoagulation	88 (16.1)	71 (15.3)	17 (20.2)	.260	547

ACEi, angiotensin-converting enzyme inhibitor; ARB, angiotensin receptor blocker; BMI, body mass index; BP, blood pressure; LVH, left ventricular hypertrophy; LVEDD, left ventricular end-diastolic diameter; LVESD, left ventricular end-systolic diameter; LVEF, left ventricular ejection fraction; NYHA, New York Heart Association; MRA, mineralocorticoid receptor antagonist; PPCM, peripartum cardiomyopathy.

### Maternal outcome

**Figure 2:**
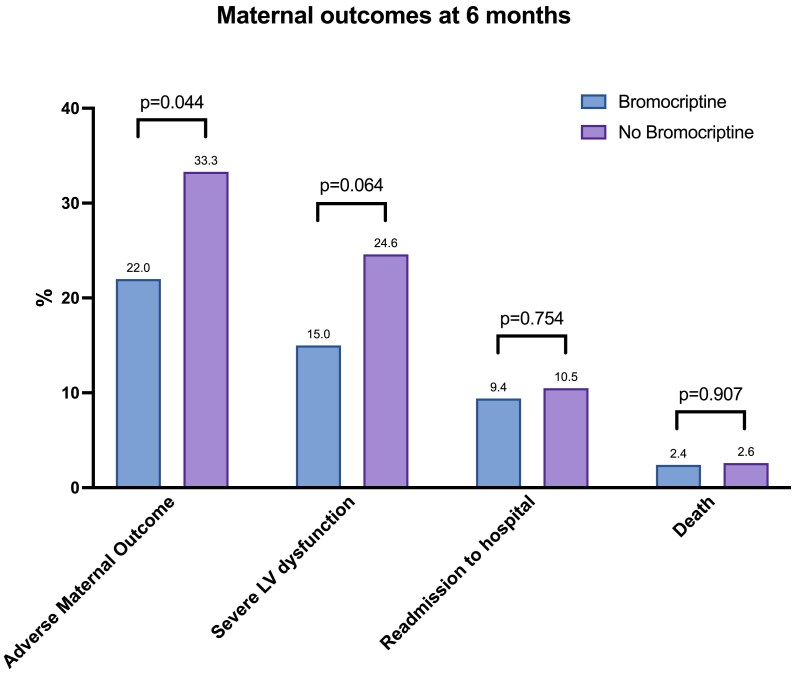
Maternal outcomes at 6 months including components of the primary endpoint as stratified by bromocriptine treatment

The primary composite endpoint was available in 491 of 552 patients (89%) and occurred in 18 out of 82 patients treated with bromocriptine (22.0%) and in 136 out of 409 patients treated with standard of care (33.3%, *P* = .044) (*Figure 2*). This was primarily driven by fewer patients with severe LV dysfunction (defined as an LVEF < 35%) at 6 months in the bromocriptine group (15.0% vs. 24.5%). The occurrence of the primary endpoint and its components are summarized in *Table 2*.

**Table 2:** Maternal outcomes at 6 months, stratified by bromocriptine treatment

	No bromocriptine (*n* = 467)	Bromocriptine (*n* = 85)	*P*-value	*n*
NYHA functional class at 6 months			.830	552
I	265 (60.6)	50 (63.3)		516
II	153 (35.0)	27 (34.2)		
III	16 (3.7)	2 (2.5)		
IV	3 (0.7)	0 (0.0)		
Systolic BP (mmHg) at 6 months	110.0 (100.0–120.0)	110.0 (104.0–120.0)	.700	
Diastolic BP (mmHg) at 6 months	70.0 (65.0–80.0)	73.0 (70.0–80.0)	.062	503
Heart rate (b.p.m.) at 6 months	76.0 (65.0–84.0)	78.0 (70.0–84.0)	.180	503
LVEDD (mm) at 6 months	54.0 (48.0–60.0)	55.0 (50.0–58.5)	.660	504
LVESD (mm) at 6 months	41.5 (35.0–50.0)	40.0 (33.0–46.0)	.140	435
LVEF (%) at 6 months	48.0 (36.0–56.0)	47.0 (42.0–55.5)	.570	381
LVEF categories at 6 months			.031	467
≤35%	95 (24.5)	12 (15.0)		
36%–49%	111 (28.7)	34 (42.5)		
≥50%	181 (46.8)	34 (42.5)		
Any thromboembolism	26 (5.6)	5 (6.0)	.900	547
Venous thromboembolism	18 (3.9)	3 (3.6)	.890	547
Arterial thromboembolism	10 (2.2)	2 (2.4)	.910	548
Ischaemic stroke	5 (1.1)	2 (2.4)	.340	549
Haemorrhagic stroke	2 (0.4)	0 (0.0)	.540	550
Any stroke	7 (1.5)	2 (2.4)	.570	549
Death within 6 months	12 (2.6)	2 (2.4)	.910	552
Readmission within 6 months	49 (10.5)	8 (9.4)	.750	550
Severe LV dysfunction at 6 months	95 (24.6)	12 (15.0)	.064	467
Severe LV dysfunction or death at 6 months	107 (26.8)	14 (17.1)	.064	481
Adverse maternal outcome at 6 months	136 (33.3)	18 (22.0)	.044	491

BP, blood pressure; LVH, left ventricular hypertrophy; LVEDD, left ventricular end-diastolic diameter; LVESD, left ventricular end-systolic diameter; LVEF, left ventricular ejection fraction; NYHA, New York Heart Association.

**Figure 3:**
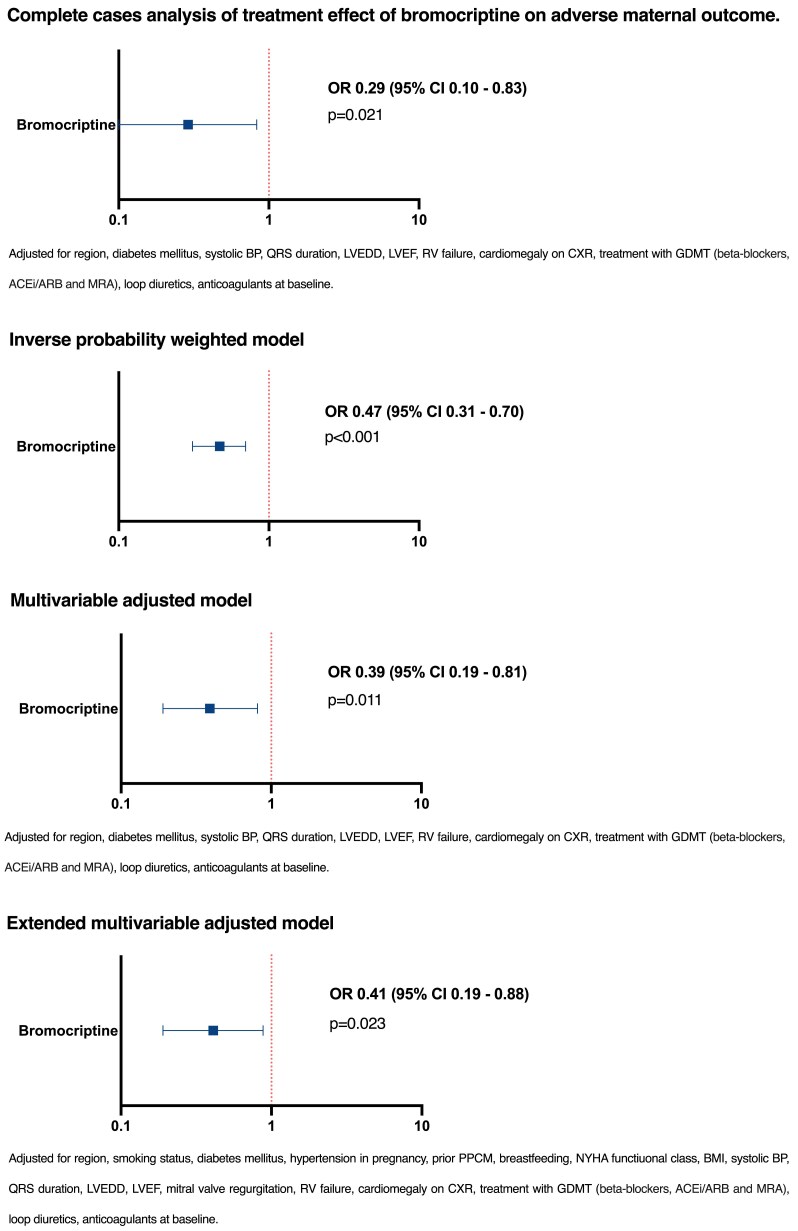
Multivariable regression analysis of the associations between bromocriptine treatment and poor maternal outcome [death, hospital readmission, or persistent severe left ventricular dysfunction (<35%) at 6-month follow-up]

In complete case analysis (*Table 3*), bromocriptine treatment, adjusted for possible confounders, was associated with reduced adverse maternal outcome [odds ratio (OR) 0.29, 95% confidence interval (CI) 0.10–0.83, *P* = .021]. This association remained after applying multiple imputation and methods to correct for confounding by indication. The estimates for bromocriptine in the inverse probability weighted model, based on the imputed data for the primary endpoint, were as follows: OR 0.47, 95% CI 0.31–0.70, *P* < 0.001. The results are summarized in *Figure 3* and *Table 3*. Thromboembolic events were observed in 6.0% of the patients in the bromocriptine group vs. 5.6% in the standard of care group (*P* = .900).

**Table 3:** Treatment effect of bromocriptine in multiple imputed data on adverse maternal outcome

Variable	Model	Weighting and adjustment	Missing data	Odds ratio (95% CI)	*P*-value
Bromocriptine treatment	1	Multivariable adjusted^a^	Complete cases (*n* = 325)	0.29 (0.10–0.83)	.021
Bromocriptine treatment	2	Inverse probability weighted model	Multiple imputed	0.47 (0.31–0.70)	<.001
Bromocriptine treatment	3	Multivariable adjusted model^a^	Multiple imputed	0.39 (0.19–0.81)	.011
Bromocriptine treatment	4	Extended multivariable adjusted model^b^	Multiple imputed	0.41 (0.19–0.88)	.023

^a^Adjusted for history of diabetes mellitus, anticoagulant treatment, treatment with GDMT (ACEi/ARB, beta-blockers, and MRAs), treatment with loop diuretics, systolic blood pressure at baseline, QRS duration at baseline, left ventricular end-diastolic diameter, left ventricular ejection fraction, region, right ventricular failure at baseline, and cardiomegaly at X-thorax at baseline.

^b^Adjusted for the variables described under ^a^ and breastfeeding in months, prior peripartum cardiomyopathy, smoking status at baseline, body mass index, hypertension in pregnancy, New York Heart Association Class at baseline, and mitral valve regurgitation at baseline.

## Discussion

### Principal findings

The principal finding of this study is that bromocriptine treatment was associated with better maternal outcome which was primarily driven by fewer patients with severe LV dysfunction after 6 months (*Structured Graphical Abstract*). Furthermore, no differences in thromboembolic events were observed in the bromocriptine group vs. the standard of care group. These data may help guide treatment decisions until evidence from randomized studies that are currently being conducted becomes available.

### Comparison with other studies

Clinical data regarding the efficacy of bromocriptine treatment in patients with PPCM remain limited. Only two randomized studies have been conducted that have provided some evidence that bromocriptine treatment might be beneficial; however, the evidence remains inconclusive due to the low number of patients that was included or the lack of a true control group in one of the studies.^8,9^ A systematic review and meta-analysis of the available evidence, including these two randomized studies and six observational studies, encompassing 593 patients with PPCM, demonstrated that bromocriptine treatment was associated with significantly higher survival rates and greater improvement in LVEF.^16^ The results of our study are consistent with these previous findings, showing that bromocriptine treatment is associated with better maternal outcomes. In the current study, this result was mainly driven by a lower number of patients with severe LV dysfunction after 6 months, despite rigorous correction for all baseline characteristics known to impact LV recovery.

Two recent studies investigated which factors are associated with an improvement in LV function in patients with PPCM. In a Scottish registry comprising of 225 women with PPCM, only LVEDD was independently associated with an improved LV function at 1 year.^17^ Similar findings were observed in the EORP PPCM registry. Here, we found that the two strongest predictors of LV recovery, defined as a LVEF ≥ 50%, were also LVEDD and, in addition to that, QRS duration.^5,18,19^ Both of these variables were comparable between patients receiving bromocriptine or no bromocriptine in our study. Another important predictor for LV recovery and maternal outcome in patients with PPCM is the development of hypertension during pregnancy. Especially the presence of pre-eclampsia is associated with a more than two-fold higher chance of LV recovery compared with women with PPCM with no gestational hypertension^20^ In our current study, the presence of pre-eclampsia was not significantly different between groups.

Loop diuretics were used in significantly more patients receiving bromocriptine indicating that these patients suffered from more severe acute heart failure/congestion than patients not treated with bromocriptine. The fact that patients in the bromocriptine group tended to be sicker than in the non-treated group indicates that bias may not explain the beneficial effects associated with bromocriptine. This is also supported by several statistical methods, used to account for confounding by indication, which all confirm the main analysis.

We did not observe a reduction in mortality or heart failure rehospitalizations in the bromocriptine group, although it must be noted that overall mortality was relatively low. Interestingly, we did not observe differences in the incidence of thromboembolic events with the use of bromocriptine. Previously, several case reports described the occurrence of myocardial infarction and retinal vein occlusion in women with PPCM also treated with bromocriptine.^21,22^ It is clear that women with PPCM similar to all women in the peripartal state are at increased risk for thromboembolic events; we have previously shown that 5% of the women with PPCM present with thromboembolism which is likely related to the hypercoagulable state, an evolutionary remnant to minimize postpartum bleeding.^23^ This in combination with LV dilatation, endothelial injury, and immobility may explain this high rate. However, our study shows that the use of bromocriptine was not associated with incident thromboembolic events. Since only 20% of the patients on bromocriptine were also using anticoagulation, our data do not support long-term treatment with oral anticoagulation in women with PPCM. However, due to the relatively low incidence of thromboembolic events during follow-up, these results alone should not inform coagulation treatment decisions.

### Strength and limitations

The study’s relatively large sample size and the methods to account for confounding by indication are its main strengths, and the results of this study may aid in bridging the gap until the results of the REBIRTH trial are published, which is expected to be in the beginning of 2029.

Several limitations must be acknowledged. First, although multiple strategies have been employed to correct for confounding by indication, these methods only allow for correction for known confounders. Therefore, there remains the risk of residual confounding due to unknown confounders. Second, patients with unknown bromocriptine status were not included in the study (*n* = 187) which introduces the risk of selection bias. Third, the results of this observational, non-randomized study should be interpreted cautiously, and further randomized studies are warranted to provide evidence of causality. However, future randomized trials will be limited by inadequate blinding as bromocriptine treatment leads to cessation of lactation.

## Conclusion

Among women with PPCM, bromocriptine treatment was associated with better maternal outcomes after 6 months and no increased risk of thromboembolic events.

## Supplementary Material

ehae559_Supplementary_Data

## Declarations

### Data Availability

Data are available upon request.
