# Supplementary material for: Bromocriptine treatment and outcomes in peripartum cardiomyopathy: the EORP PPCM registry
Source: Eur Heart J. 2024 Sep 2;46(11):1017–27. doi: 10.1093/eurheartj/ehae559 (PMC11905762; doi:10.1093/eurheartj/ehae559)
Supplement: ehae559_Supplementary_Data [file ehae559_supplementary_data.pdf]

Appendix 1 - PPCM

EORP Oversight Committee

C.P. Gale, GB (Chair); B. Beleslin, RS; A. Budaj, PL; O. Chioncel, RO; N. Dagres, DE; N. Danchin, FR; J. Emberson, GB; D. Erlinge, SE; M. Glikson, IL; A. Gray, GB; M. Kayikcioglu, TR; A.P. Maggioni, IT; V.K. Nagy, HU; A. Nedoshivin, RU; A-S. Petronio, IT; J. Roos-Hesselink, NL; L. Wallentin, SE; U. Zeymer, DE.

Executive Committee

J. Bauersachs, DE (Chair); K. Sliwa, ZA (Chair); M. Boehm, DE; M Johnson, GB; D. Hilfiker-Kleiner, DE; A.P. Maggioni, IT; A. Mbakwem, NG; A. Mebazaa, FR; F. Mouquet, FR; M. Petrie, GB; B. Pieske, DE; V. Regitz-Zagrosek, DE; J. Roos-Hesselink, NL; M. Schaufelberger, SE; P.M. Seferovic, RS; L. Tavazzi, IT; P. van der Meer, NL; K. Van Spaendonck-Zwarts, NL.

Investigators

Argentina: *Buenos Aires:* R. Favaloro, L. Favaloro, M. Carballo, L. Favaloro, M. Peradejordi, M.F. Renedo, D. Absi, A. Bertolotti, R. Ratto, M.L. Talavera, R. Gomez, **Australia:** *Clayton:* S. Lockwood, T. Barton, M-A. Austin, *Elizabeth Vale:* M. Arstall, E. Aldridge, Y.Y. Chow, G. Dekker, G. Mahadavan, J. Rose, M. Wittwer, **Austria:** *Salzburg:* U. Hoppe, A. Sandhofer, **Azerbaijan:** *Baku:* A. Bahshaliyev, Z. Gasimov, A. Babayev, P. Niftiyev, I. Hasanova, **Bahrain:** *Manama:* R. AlBannay, W. AlHaiki, A. Husain, N. Mahdi, **Belarus:** *Minsk:* A. Kurlianskaya, M. Lukyanchyk, O. Shatova, T. Troyanova-Shchutskaia, **Belgium:** *Brussels:* L. Anghel, *Gent:* M. De Pauw, S. Gevaert, J. De Backer, M. De Hosson, P. Vervaet, *Hasselt:* P.J. Timmermans, A. Janssen, **Burkina Faso:** *Ouagadougou:* N.V. Yameogo, L.J. Kagambega, **Canada:** *Sherbrooke:* A. Cumyn, N. Caron, A-M. Cote, N. Sauve, **Congo, Republic Of:** *Lubumbashi:* D. Ngoy Nkulu, D. Malamba Lez, E. Ngoy Yolola, **Czech Republic:** *Brno:* J. Krejci, H. Poloczkova, **Denmark:** *Copenhagen:* A. Ersboll, F. Gustafsson, **Egypt:** *Alexandria:* Y. Elrakshy, *Alexandria:* M. Hassanein, B. Hammad, O. Nour Eldin, *Assiut:* D. Fouad, S. Salman, Z. Zareh, D. Abdeall, *Benha:* H. Abo Elenin, H. Ebaid, A. El Nagar, S. Farag, M. Saed, Dr Y H Abd El Rahman, *Cairo:* B.S. Ibrahim, *Cairo:* M. Abdelhamid, R.N. W. Hanna, G. Youssef, R. Awad, O.L. I. Botrous, S. Ibrahim Halawa, *Ismailya:* G. Nasr, *Zagazig:* A. Saad, *Zagazig:* M. El Tahlawi, *Zagazig:* M. Abdelbaset, M. El-saadawy, A. El-shorbagy, G. Shalaby, **Finland:** *Lahti:* O. Anttonen, H. Tolppanen, S. Hamekoski, **France:** *Brest:* T. Menez, A. Noel, *Lille:* N. Lamblin, F. Mouquet, C. Coulon, P. de Groote, S. Langlois, G. Schurtz, *Paris:* A. Cohen-Solal, A. Mebazaa, M-C. Fournier, B. Louadah, N. Akrout, D. Logeart, *Rennes:* G. Leurent, **FYR Macedonia:** *Skopje:* S. Jovanova, F. Arnaudova-Dezulovicj, V. Livrinova, **Germany:** *Hannover:* J. Bauersachs, D. Hilfiker-Kleiner, D. Berliner, M. Jungesblut, T. Koenig, V.A. Moulig, T.J. Pfeffer, *Homburg/Saar:* M. Böhm, I. Kindermann, V. Schwarz, *Karlsruhe:* C. Schmitt, P. Swojanowsky, **Great Britain:** *Cambridge:* S. Pettit, *Glasgow (Scotland):* M. Petrie, M. McAdam, D. Patton, *Herts:* A. Bakhai, V. Krishnamurthy, L. Lim, *High Wycombe:* P. Clifford, N. Bowers, *Hull:* A. L. Clark, *Leeds:* K. Witte, D. Cullington, J. Oliver, A. Simms, M. Mcginlay, *London:* T. McDonagh, A. M. Shah, G. Amin-Youssef, J. De Courcey, K. Martin, *Manchester:* S. Shaw, *Manchester:* S. Vause, *Nottingham:* S. Wallace, G. Malin, *Poole, Dorset:* C. Wick, **Greece:** *Athens:* M. Nikolaou, I. Rentoukas, **Honduras:** *Tegucigalpa:* H. Chinchilla, L.

Andino, **India:** *Bangalore:* S. Iyengar, S. Chandra V, D.K. Yadav, R. Ravi Babu, *Bokaro Steel City:* A.K. Singh, S. Kumar, B.B. Karunamay, S.K. Chaubey, S.R. Dhiman, V.C. Jha, S.K. Singh, *Hyderabad:* D. Kodati, R. Dasari, S. Sultana, **Indonesia:** *Bandung:* T.I. Dewi, H. Sasmaya Prameswari,
Iraq: *Bagdad:* H.A. Al-Farhan, A. Al-Hussein, I.F. Yaseen, Falah Al-Azzawi, Ghazi Al-Saedi, G.M. Mahmood, M.K. Mohammed, A.F. Ridha, **Israel:** *Hadera:* A. Shotan, A. Vazan , *Rehovot:* S. Goland, M. Biener,
Italy: *Bergamo:* M. Senni, A. Grosu, E. Martin, *Bologna:* D. Degli Esposti, S. Bacchelli, C. Borghi , *Brescia:* M. Metra, E. Sciatti, R. Orabona, *Florence:* F. Sani, *Foggia:* N.D. Brunetti, *Trieste:* G. Sinagra, M. Bobbo, B. D'Agata Mottolese, V. Gesuete, S. Rakar, F. Ramani,
Japan: *Osaka:* C. Kamiya, **Kenya:** *Nairobi:* A. Barasa, M. Ngunga, **Kosovo:** *Prishtina:* G. Bajraktari, V. Hyseni, D. Lleshi, E. Pllana, T. Pllana, **Kyrgyzstan:** *Bishkek:* A. Noruzbaeva, F. Ismailov, *Bishkek:* E. Mirrakhimov, S. Abilova, O. Lunegova, *Bishkek:* A. Kerimkulova, G. Osmankulova, M. Duishenalieva, B. Kurmanbekova, M. Turgunov, S. Mamasaidova, E. Bektasheva,
Lithuania: *Kaunas:* A. Kavoliuniene, G. Muckiene, A. Vaitiekiene, *Vilnius:* J. Celutkiene, L. Balkevicine, J. Barysiene, **Malaysia:** *Kuala Lumpur:* K.H. Chee, **Mozambique:** *Maputo:* A. Damasceno, M. Machava,
Netherlands: *Groningen:* D.J. van Veldhuisen, P. van der Meer, M. van den Berg, *Rotterdam:* J. Roos-Hesselink, I. van Hagen, L. Baris, **Nicaragua:** *Managua:* P. Hurtado,
Nigeria: *Abakaliki:* P. Ezeonu, G. Isiguzo, N. Obeka, R. Onoh, F. Asogwa, C. Onyema, K. Otti, *Abuja:* D. Ojji, A. Odili, A. Nwankwo, *Kano:* K. Karaye, N. Ishaq, B. Sanni, H. Abubakar, B. Mohammed, *Kano State:* M. Sani, *Mushin, Lagos:* M. Kehinde, A. Mbakwem, B. Afolabi, C. Amadi, M. Kilasho,
Pakistan: *Karachi:* N. Qamar, S. Furnaz, S. Gurmani, *Rawalpindi:* M.G.A. Mahmood Kayani, R. Munir, S. Hussain, S. Malik, S. Mumtaz, **Philippines:** *Ozamiz City:* J.R. Saligan, **Poland:** *Krakow:* P. Rubis, B. Biernacka-Fijalkowska, A. Lesniak-Sobelga, S. Wisniowska-Smialek, *Lodz:* J.D. Kasprzak, *Lodz:* M. Lelonek, P. Zycinski, L. Jankowski, *Poznan:* S. Grajek, Z. Oko-Sarnowska, A. Bartczak Rutkowska, M. Kaluzna-Oleksy, K. Plaskota, *Warszawa:* M. Demkow, Z. Dzielinska, J. Henzel, K. Kryczka,
Russian Federation: *Saint Petersburg:* O. Moiseeva, O. Irtyuga, E. Karelkina, I. Zazerskaya, **Serbia:** *Belgrade:* P.M. Seferovic, I. Milinkovic, I. Živkovic, A.D. Ristic, D. Milasinovic, **Singapore:** *Singapore:* W. KF Kong, *Singapore:* L.K. Tan, J.L. Tan, S. Thain, K.K. Poh, J. Yip, **South Africa:** *Cape town:* K. Sliwa, F. Azibani, J. Hovelmann, C. Viljoen, O. Briton, **Spain:** *Badalona:* E. Zamora, *Leon:* N. Alonso Orcajo, R. Carbonell, C. Pascual, *Madrid:* J. Farre Muncharaz, *Madrid:* L. Alonso-Pulpon, J. Segovia Cubero, M. Taibo Urquia, P. Garcia-Pavia, M. Gomez-Bueno, M. Cobo-Marcos, A. Briceno, *Malaga:* E. De Teresa Galvan, J.M. Garcia-Pinilla, A. Robles-Mezcua, L. Morcillo-Hildalgo,
Sudan: *Khartoum:* A. Elbushi, A. Suliman, N. Ahamed, K. Jazzar, M. Murtada, **Sweden:** *Goteborg:* M. Schaufelberger, V. Goloskokova, **Switzerland:** *Lausanne:* R. Hullin, N. Yarol, *Zurich:* M. Arrigo,
Turkey: *Eskisehir:* Y. Cavusoglu, S. Eraslan, *Istanbul:* A.S. Fak, *Istanbul:* S. Catirli Enar, L. Sarac, B. Cankurtaran, *VAN:* H. Gumrukcuoglu, F. Ozturk, **Uganda:** *Kampala:* J. Omagino, *Kampala:* C. Mondo, P. Lwabi, P. Ingabire, J. Nabbaale, W. Nyakoojo, E. Okello, E. Sebatta, I. Ssinabulya, E. Atukunda, S. Kitooleko, T. Semu, **United Arab Emirates:** *Abu Dhabi:* B.T. Salih, *Abu Dhabi:* A.M. Komaranchath, W.A.R. Almahmeed, F. Gerges, F.S. Mohamed Farook, F. Albakshy, N. Mahmood, S. Wani,
United States: *Allentown:* R. Freudenberger, N. Islam, J. Quinones, D. Sundlof, C. Beitler, L. Centolanza, K. Cornell, S. Huffaker, L. Matos, *Mineola, NY:* K. Marzo, V. Paruchuri, D. Patel, **Uzbekistan:** *Tashkent:* T. Abdullaev, *Tashkent:* B. Alyavi, S. Mirzarakhimova, I. Tsoy, R. Bekbulatova, J. Uzokov.
